# EFF_D_SVM: a robust multi-type brain tumor classification system

**DOI:** 10.3389/fnins.2023.1269100

**Published:** 2023-09-29

**Authors:** Jincan Zhang, Xinghua Tan, Wenna Chen, Ganqin Du, Qizhi Fu, Hongri Zhang, Hongwei Jiang

**Affiliations:** ^1^College of Information Engineering, Henan University of Science and Technology, Luoyang, China; ^2^The First Affiliated Hospital, and College of Clinical Medicine of Henan University of Science and Technology, Luoyang, China

**Keywords:** brain tumors, transfer learning, feature extraction, grad-CAM, robustness

## Abstract

Brain tumors are one of the most threatening diseases to human health. Accurate identification of the type of brain tumor is essential for patients and doctors. An automated brain tumor diagnosis system based on Magnetic Resonance Imaging (MRI) can help doctors to identify the type of tumor and reduce their workload, so it is vital to improve the performance of such systems. Due to the challenge of collecting sufficient data on brain tumors, utilizing pre-trained Convolutional Neural Network (CNN) models for brain tumors classification is a feasible approach. The study proposes a novel brain tumor classification system, called EFF_D_SVM, which is developed on the basic of pre-trained EfficientNetB0 model. Firstly, a new feature extraction module EFF_D was proposed, in which the classification layer of EfficientNetB0 was replaced with two dropout layers and two dense layers. Secondly, the EFF_D model was fine-tuned using Softmax, and then features of brain tumor images were extracted using the fine-tuned EFF_D. Finally, the features were classified using Support Vector Machine (SVM). In order to verify the effectiveness of the proposed brain tumor classification system, a series of comparative experiments were carried out. Moreover, to understand the extracted features of the brain tumor images, Grad-CAM technology was used to visualize the proposed model. Furthermore, cross-validation was conducted to verify the robustness of the proposed model. The evaluation metrics including accuracy, F1-score, recall, and precision were used to evaluate proposed system performance. The experimental results indicate that the proposed model is superior to other state-of-the-art models.

## 1. Introduction

Brain tumors pose a serious threat to people’s health and have a high fatality rate (Alyami et al., 2023). Early detection of brain tumors is crucial for patients, as they can get a greater chance of survival (Özbay and Altunbey Özbay, 2023). Medical imaging techniques have been widely used by radiologists. Among these techniques, Magnetic Resonance Imaging (MRI) is one of the most common techniques for diagnosing and evaluating brain tumors, which could provide rich brain tissue data (Gu and Li, 2021; Ayadi et al., 2022). However, the traditional MRI detection of brain tumors heavily relies on experienced doctors. Fatigue caused by prolonged working hours could affect doctor diagnosis, resulting in potential risks to patients. Therefore, it is necessary to develop an automated brain tumor classification computer-aided system to assist doctors in diagnosis (Nanda et al., 2023).

Brain tumors are commonly classified as either benign or malignant, with malignant tumors being further classified into three subtypes: glioma tumor, pituitary tumor, and meningioma tumor. Classifying brain tumors into multiple categories is more challenging than classifying them into two categories (Gu et al., 2021; Shahin et al., 2023).

Machine learning and deep learning are widely used in cancers study (Maurya et al., 2023). Typical ML classification methods encompass a series of steps: data preprocessing, feature extraction, feature selection, dimensionality reduction, and classification (Swati et al., 2019). Bi et al. (2021) and Saravanan et al. (2020) have both utilized machine learning to achieve the task of classifying skin cancers. Bi et al. (2021) utilized a combination of Support Vector Machine (SVM) and Chaotic World Cup Optimization (CWCO) optimization algorithms, whereas Saravanan et al. (2020) used SVM as a classifier and Gray-Level Co-Occurrence Matrix (GLCM) for feature extraction. Amin et al. (2020) employed SVM for brain tumors Classification. Feature extraction is a key step in achieving high performance in traditional machine learning. The accuracy of classification often depends on the features extracted with the help of experts. However, for most researchers, feature extraction is a challenging task when using traditional machine learning methods in research. The applications of machine learning and deep learning in disease classification are introduced in this paper.

In machine learning, it is necessary to perform feature extraction. Cheng et al. (2015) utilized three feature extraction techniques, namely intensity histogram, grey-scale co-occurrence matrix, and bag-of-words, achieving a model accuracy of 91.28%. Gumaei et al. (2019) employed a hybrid feature extraction approach to extract brain tumor images feature, which was combined with a regularized extreme learning machine for the classification of brain tumors, and an accuracy of 94.233% on the Chen dataset was achieved. Khan et al. (2019) used the watershed algorithm for image segmentation in a brain tumor classification system. The brain tumor classification system categorized tumors as either benign or malignant with an accuracy of 98.88%.

Since dataset features can be automatically extracted by deep learning techniques, they have got more and more attention (Bar et al., 2015). As a deep learning technique, Convolutional Neural Network (CNN) models have been widely used in the field of deep learning for tasks such as image classification, object detection, and face recognition. CNN models are mainly composed of convolutional layers, pooling layers, and fully connected layers. Convolutional layers use filters to perform convolution operations on input data and extract features of images. Pooling layers are used to downsample the features outputted by convolutional layers, reducing the number of features and parameters. The fully connected layer connects the output of the pooling layer to the final output layer for tasks such as classification or regression. Unlike traditional machine learning techniques, the CNN model can automatically learn useful features from images, eliminating the need for manual feature engineering, so it is an ideal choice for medical image processing (Yu et al., 2022; Maurya et al., 2023). Medical image datasets are generally small due to the difficulty and cost of acquisition. Therefore, as an effective small dataset processing technology, transfer learning has been widely applied in the field of medical image classification such as breast cancer, pneumonia, brain tumors, and glomerular disease (Yu et al., 2022). Talo et al. (2019) categorized brain tumors as benign or malignant using the pre-trained RestNet34. Kaur and Gandhi (2020) used pre-trained models such as Resnet50 and GoogLeNet ResnNet101 to classify brain tumors. Deepak and Ameer (2019) introduced a method using pre-trained GoogLeNet. Fine-tuned GoogLeNet was used to extract features of brain tumor images, and then SVM and KNN were employed as classifiers to complete the brain tumor classification task. EfficientNets, as lightweight models, are also extensively utilized in applications such as brain tumor classification (Tan and Le, 2019). Shah et al. (2022) used the EfficientNetB0 model to classify brain tumors as healthy and unhealthy. Nayak et al. (2022) utilized EfficientNetB0 to perform a triple classification of brain tumors, while Zulfiqar et al. (2023) utilized EfficientNetB2 for the same task. Yet, the model proposed in (Nayak et al., 2022) suffered from mild overfitting, resulting in low classification accuracy. And Zulfiqar et al. (2023) achieved a classification accuracy of only 91.35% when performing cross-validation experiments on different datasets. Additionally, Nayak et al. (2022) and Zulfiqar et al. (2023) only performed triple classification task of brain tumors.

Abiwinanda et al. (2019) created a model consisting of two convolution layers, an activation-Relu layer, and a Dense-64 layer. The model achieved an accuracy rate of 84.19%. Alanazi et al. (2022) constructed a 22-layer CNN architecture. The model was trained using a large-scale binary classification dataset, and then it was fine-tuned using a transfer learning approach. The accuracy of the model got 96.89 and 95.75% for Chen and Kaggle datasets, respectively. Kibriya et al. (2022) proposed a 13-layer CNN model and achieved 97.2 and 96.9% accuracy on Chen and Kaggle data sets. Jaspin and Selvan (2023) presented a 10-layer model using different optimizers (Adam and RMSprop) to train the model. On the Chen dataset, the accuracy of 96% was obtained using Adam and 95% was achieved using RMSprop. The studies by Swati et al. (2019) and Rehman et al. (2020) utilized the VGG19 and VGG16 models, respectively, and achieved accuracy rates of 94.82 and 98.69%. Sajjad et al. (2019) segmented the brain tumor region and used VGG19 for image classification, achieving an accuracy of 94.58%. Ghassemi et al. (2020) performed a brain tumor classification task based on a pre-trained Generative Adversarial Network (GAN) with an accuracy of 95.6%. Satyanarayana (2023) combined convolutional neural networks with a deep learning approach based on mass correlation and reported a classification accuracy of 94%. The proposed framework involved the construction of a multi-task CNN model and a 3D densely connected convolutional network. The authors combined the features extracted from a multi-task CNN and a 3D densely connected convolutional network to classify Alzheimer’s disease.

Moreover, it has been proven that combining pre-trained models with machine learning is also a feasible method. Kang et al. (2021) used MobileNetV2 to extract features from brain MRI images, and adopted the SVM algorithm for classification, obtaining an accuracy of 91.58%. In reference (Sekhar et al., 2022), MobileNetV2 was used to extract features from brain tumor images. The extracted features were then classified using SVM and K-Nearest Neighbors (KNN). The best classification accuracy of 98.3% is achieved using KNN. Öksüz et al. (2022) utilized ResNet18 to extract both shallow and deep features from an enlarged Region of Interest (ROI) in brain tumors.

By integrating the shallow and deep features, a classification of the tumors was carried out using SVM and KNN classifiers. The results indicated an overall classification accuracy of 97.25% with the SVM classifier and 97.0% with the KNN classifier. Demir and Akbulut (2022) proposed a new model, in which an R-CNN (Residual-CNN) structure was designed to extract features, using SVM as the classifier, with an accuracy of 96.6% being obtained. Deepak and Ameer (2023) used an additive loss function to train the CNN model, updating the model using different optimizers, then combined it with SVM and finally voted the classification results to derive the final classification result. The model obtained an accuracy of 95.6%. Muezzinoglu et al. (2023) built a new framework PatchResNet. Firstly, using a pre-trained ResNet50 to extract features from same-sized image blocks, feature selection was performed over Neighborhood Component Analysis (NCA), Chi2, and ReliefF. Secondly, the features were fed into the classifier KNN. Finally, majority voting was used to obtain the final prediction result with an accuracy rate of 98.1%.

Optimization algorithms have also been utilized to improve the performance of brain tumor classification systems. In reference (Kabir Anaraki et al., 2019), a Genetic Algorithm (GA) was used to optimize the CNN structure and achieved 94.2% accuracy. Kumar and Mankame (2020) combined the dolphin echolocation algorithm with the Sine Cosine Algorithm (SCA) to segment brain tumors from MRI and used the segmented images for brain tumor classification. Mehnatkesh et al. (2023) applied Improved Ant Colony Optimization (IACO) to optimize the super parameters of the ResNet architecture for brain tumor classification, achieving a classification accuracy rate of 98.694%.

The preceding discussion highlights the extensive adoption of deep learning as a prevalent technique for brain tumor classification. Nevertheless, the optimization of network structures using algorithmic approaches is time-intensive. Training the network from the ground up demands a substantial dataset and entails lengthier training compared to migration-based learning approaches. Furthermore, most of the prior studies have only employed a single dataset without conducting cross-dataset validation. However, our work utilized a pre-trained CNN model and incorporated regularization techniques to combat overfitting. The classification of brain tumors was successfully accomplished by the incorporation of machine learning techniques. Moreover, to verify the generalization performance of the proposed model, some experiments were carried out using two publicly available datasets while performing cross-data validation. And by adding Gaussian noise and salt-and-pepper (S&P) noise to the pictures of the brain tumor, the robustness of the model was further demonstrated.

We presented a novel feature extraction module based on EfficientNetB0 and employed SVM to categorize the resultant features. Specifically, we evaluated the model performance using both triple classification (glioma tumor, meningioma tumor, and pituitary tumor) and quadruple classification (glioma tumor, meningioma tumor, pituitary tumor, and healthy), providing comprehensive validation for our proposed model. In this paper, we presented an automated classification model of brain tumors, and the model was evaluated on two publicly available datasets (Chen and Kaggle). The model used a pre-trained EfficientNetB0 CNN model and combined dropout regularization and dense layers to construct a new feature extraction module EFF_D. The highest classification performance was achieved using the SVM classifier. The main research contributions of this study are as follows:

1. A new model is proposed for brain tumor classification.
2. Based on two public datasets, the proposed model has been proven to be a reliable method for brain tumor classification.
3. By using the last convolution layer of the Grad-CAM visualization model, a localized heat map was obtained, highlighting the brain tumor region.
4. The proposed model can classify brain tumors better than the available models. And the cross-data validation of the model achieves better result.

## 2. Materials and methods

This section focuses on our proposed approach. The base model used in this method is the pre-trained EfficientNetB0. Firstly, Relevant dropout and dense layers were introduced to construct a new model. Secondly, optimal hyperparameters were utilized to train the new model. Finally, the trained model was subsequently used to extract intricate image features, which were then classified utilizing the SVM algorithm. This approach is helpful in achieving better results in brain tumor classification tasks.

### 2.1. Introduction to the EfficientNetB0

EfficientNets is a series of convolutional neural network architectures developed by the Google team, making creative use of compound scaling. Of these, EfficientNetB0, as the base model, primarily consists of 16 mobile inverted bottleneck convolution (MBConv) modules (Tan and Le, 2019). In addition, the EfficientNetB0 architecture was utilized to perform 1,000 image classifications on the ImageNet dataset. According to the TensorFlow website^1^, input images for the model should be represented as floating-point tensors with three color channels and pixel values ranging from 0 to 255.

### 2.2. Datasets and preprocessing

The experiments were performed on two publicly available brain tumor datasets. The Chen dataset is the CE-MRI dataset shared by Cheng et al. (2015), which consists of 3,064 brain MRI images from 233 patients, including three types of brain tumors, namely glioma, meningioma, and pituitary tumors. The number of images of the three types of brain tumors in the dataset is 1,426, 708, and 930. The Kaggle dataset was obtained from Kaggle (Bhuvaji et al., 2020), which is comprised of 3,264 images including four categories: glioma, meningioma, pituitary, and healthy. The number of images of the four categories in the Kaggle dataset is 926, 937, 901, and 500.

The image of the Chen dataset has a size of 512 × 512 and is a grayscale image. Therefore, the image of Chen needs to be resized to 224 × 224 × 3. The image sizes in the Kaggle dataset are inconsistent, with some grayscale images and some RGB images. Similarly, the images should be adjusted to a uniform size of 224 × 224 × 3. In this paper, the data is randomly divided into non-overlapping training and test sets. The training set comprises 80% of the total dataset, while the remaining 20% is allocated to the test set.

### 2.3. Classification system

Both the datasets employed in this study, the Chen dataset, which has a total of 3,064 photos, and the Kaggle dataset, which has a total of 3,261 images—are tiny, making migration learning an effective method. The method of transfer learning is frequently used to train neural networks on a small dataset. In general, the process of training neural networks requires large dataset, but the number of brain tumor samples available is limited (Shin et al., 2016; Swati et al., 2019; Yu et al., 2022). Transfer learning offers an effective remedy for small sample size issues by enabling a transfer of knowledge from relevant tasks to new ones. Moreover, application of trained weights enhances both the efficiency and accuracy of models.

**Figure 1:**
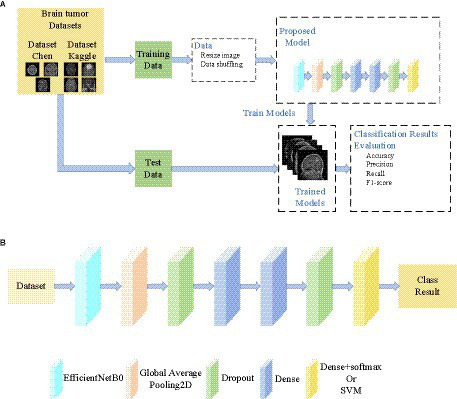
General structure of the paper **(A)** Framework of the proposed brain tumor classification system **(B)** The proposed model.

The overall architecture and method proposed in this paper are shown in Figure 1. The framework of the proposed brain tumor classification system is shown in Figure 1A. The dataset is divided into a training set and a test set, and they do not cross each other. The proposed model was trained on the training set, and the resulting trained model was saved to disk. The saved model was applied to classify the test set, and its performance was evaluated. As shown in Figure 1B, EfficientNetB0 is utilized as the foundation of our model. Table 1 describes the detailed parameters of the proposed model. The EfficientNetB0 model achieved high accuracy in classification tasks and was pre-trained on the large-scale ImageNet dataset (Tan and Le, 2019). As the dataset used in this experiment differs from the ImageNet dataset, the classification layer of the pre-trained model was removed. Then, we added two layers of Dropout to prevent overfitting, as well as two layers of Dense and one layer of Dense+Softmax to enable the model to classify our target images. The dropout ratios are 0.345 and 0.183, respectively, and the number of neurons in the Dense layer are both 69. The number of neurons in the Classification layer are either 3 or 4. When using an SVM as a classifier, the features extracted from the last Dropout layer can be used for SVM classification. The feature extraction module is called EFF_D, where the method using the Softmax classifier is called EFF_D_Softmax and the method using the SVM is called EFF_D_SVM.

**Table 1:** Parameters of the proposed model.

Model	Parameters	Setting
EFF_D_Softmax	Dropout_1	0.345
	Dense_1	69
	Dense_2	69
	Dropout_2	0.183
	optimizer	Adam
	Learning rate	0.001
	Batch size	16
	Loss function	Cross entropy
	epoch	25
EFF_D_SVM	C	1
	kernel	linear
	probability	True

### 2.4. Training CNNs

The training of a convolutional neural network combines forward and backward propagation. It starts at the input layer and is propagated forward. Then, the loss is back propagated to the first layer. In layer l, the i-th neuron receives the input from neuron j in layer l-1 through a computation process. Training samples xj are weighted by Eq. 1.


(1) $$In=\overset{n}{\underset{j=1}{\sum }}W_{\mathrm{ij}}^{l}x_{j}+b_{i}$$

where, ${}^{W_{ij}^{l}}$ represents weights, *b*_i_ denotes bias. After computing the weighted sum of the variables (*In*), the resulting values are processed through the activation functions: Swish and Relu, as represented by Eqs 2, 3, respectively.


(2) $$S_{i}^{l}=In_{i}^{l}\times sigmoid\left(\beta In_{i}^{l}\right)$$

(3) $$R_{i}^{l}=\max\left(0,In_{i}^{l}\right)$$

here, ${}^{S_{i}^{l}}$ is the output using Swish, and *β* is a constant. ${}^{R_{i}^{l}}$ is the output using Relu. The neurons in both the convolutional and fully connected layers are calculated using Eqs 1, 2 (or 3). The classification layer is calculated using the Softmax function which is shown as Eq. 4.


(4) $$y_{i}=\frac{\exp\left(x_{i}\right)}{\sum_{j}^{k}\exp\left(x_{j}\right)}$$

where, *K* is the number of categories, *x*_i_ is the i-th element of the input vector x, and *y*_i_ is the i-th element of the output vector y.

The cross-entropy loss function evaluates the prediction error of the model by comparing the predicted probability distribution generated by the model with the distribution of the true labels, as represented by Eq. 5. This loss function is utilized in the backpropagation process to optimize the model’s parameters and enhance the accuracy of the prediction results.


(5) $$L=\frac{-1}{m}\overset{m}{\underset{i}{\sum }}\ln\left(p\left(\frac{y_{i}}{x_{i}}\right)\right)$$

here, *m* represents the total number of samples, *x_i_* indicates the training sample indexed i, *y*_i_ represents the corresponding label of *x*_i_, and P denotes the probability that *x*_i_ belongs to class *y*_i_.

The model weights are updated according to Eq. 6.


$$\gamma^{t}=\gamma^{\left[\frac{\mathrm{tN}}{m}\right]}$$

(6) $$V^{t+1}=\mu V_{l}^{t}-\gamma^{t}\alpha_{l}\frac{\partial C}{\partial W}$$

$$W_{i}^{t+1}=W_{l}^{t}+V_{t}^{t+1}$$

where, *α*_l_, *γ*^t^ and μ represent different factors affecting the current iteration of the learning algorithm. *α*_l_ corresponds to the learning rate at layer l. *γ*^t^ represents the scheduling rate which reduces the initial learning rate and *μ* is used to describe the influence of previously updated weights on the current iteration.

## 3. Results and discussion

The experiments were performed in Win11 operating system with 16 G RAM and RTX3060 graphics card of 6 G video memory.

### 3.1. Performance evaluation

The dataset exhibits an imbalance, thus, it is insufficient to only accuracy is used to quantify model performance. Except for accuracy, precision, recall, and F1-score metrics are also utilized to evaluate the model performance (Alsaggaf et al., 2020). The calculation formulas for these metrics are expressed as follows:


(7) $$\mathrm{Accuracy}=\frac{\mathrm{TP}+\mathrm{TN}}{\mathrm{TP}+\mathrm{TN}+\mathrm{FP}+\mathrm{FN}}$$

(8) $$\mathrm{Precision}=\frac{\mathrm{TP}}{\mathrm{TP}+\mathrm{FP}}$$

(9) Recall=TPTP+FN

(10) $$F1-\mathrm{score}=\frac{2\times \mathrm{precision}\times \mathrm{recall}}{\mathrm{precision}+\mathrm{recall}}$$

where, TP (True Positive) is the number of correct positive predictions, TN (True Negative) is the number of correct negative predictions, FP (False Positive) is the number of wrong positive predictions, and FN (False Negative) is the number of wrong negative predictions.

### 3.2. The selection of the benchmark model

The paper conducts a comparative analysis of VGG19, ResNet50, DenseNet121, and EfficientNetB0 models in relation to training time, inference time, total parameters, and test set accuracy. Each model is compared using the Chen dataset, which has seen extensive used. Inference time represents the time required to predict 612 images from the test set. The outcomes of the experiments are presented in Table 2. Although fine-tuning EfficientNetB0 takes relatively more time, its inference time is also faster. Furthermore, EfficientNetB0 has the highest classification accuracy. Therefore, EfficientNetB0 is chosen as the benchmark model.

**Table 2:** Comparison of benchmark models.

Model	Training time (seconds)	Inference time (seconds)	Parameters (million)	Test accuracy(%)
VGG19	542	7	20.03	87.09
ResNet50	397	4	23.59	91.18
DenseNet121	524	3	7.04	96.57
EfficientNetB0	500	4	4.05	98.37

### 3.3. Experimental results

In order to further verify the effectiveness of the proposed model, a series of comparison models were also designed in this article. Initially, the neuron count in EfficientNetB0’s classification layer is aligned with the number of categories in the dataset used for classification. The model is then subjected to fine-tuning. The model employing the Softmax classifier is referred to as EFF_Softmax, while the one employing the SVM classifier is labeled as EFF_SVM.

The training steps of the proposed EFF_D_SVM model are as follows:

*Step 1*: Importing the data and resizing the images to split the data into a training set and a test set.

*Step 2*: Loading the model and pre-trained weights, removing the Top layer, and adding the Dropout and Dense layers.

*Step 3*: Training EFF_D_Softmax to classify brain tumor images.

*Step 4*: Using EFF_D to extract features and using SVM to classify brain tumors.

Similarly, the same steps are adopted to train EFF_Softmax and EFF_SVM.

The experiments were performed using two datasets. The dataset Chen was used for testing 612 images consisting of 285 glioma tumor images, 141 meningioma tumor images and 186 pituitary tumor images. The Kaggle was used for testing 652 images including 185 glioma tumor images, 187 meningioma tumor images, 180 pituitary tumor image, and 100 no-tumor images.

**Figure 2:**
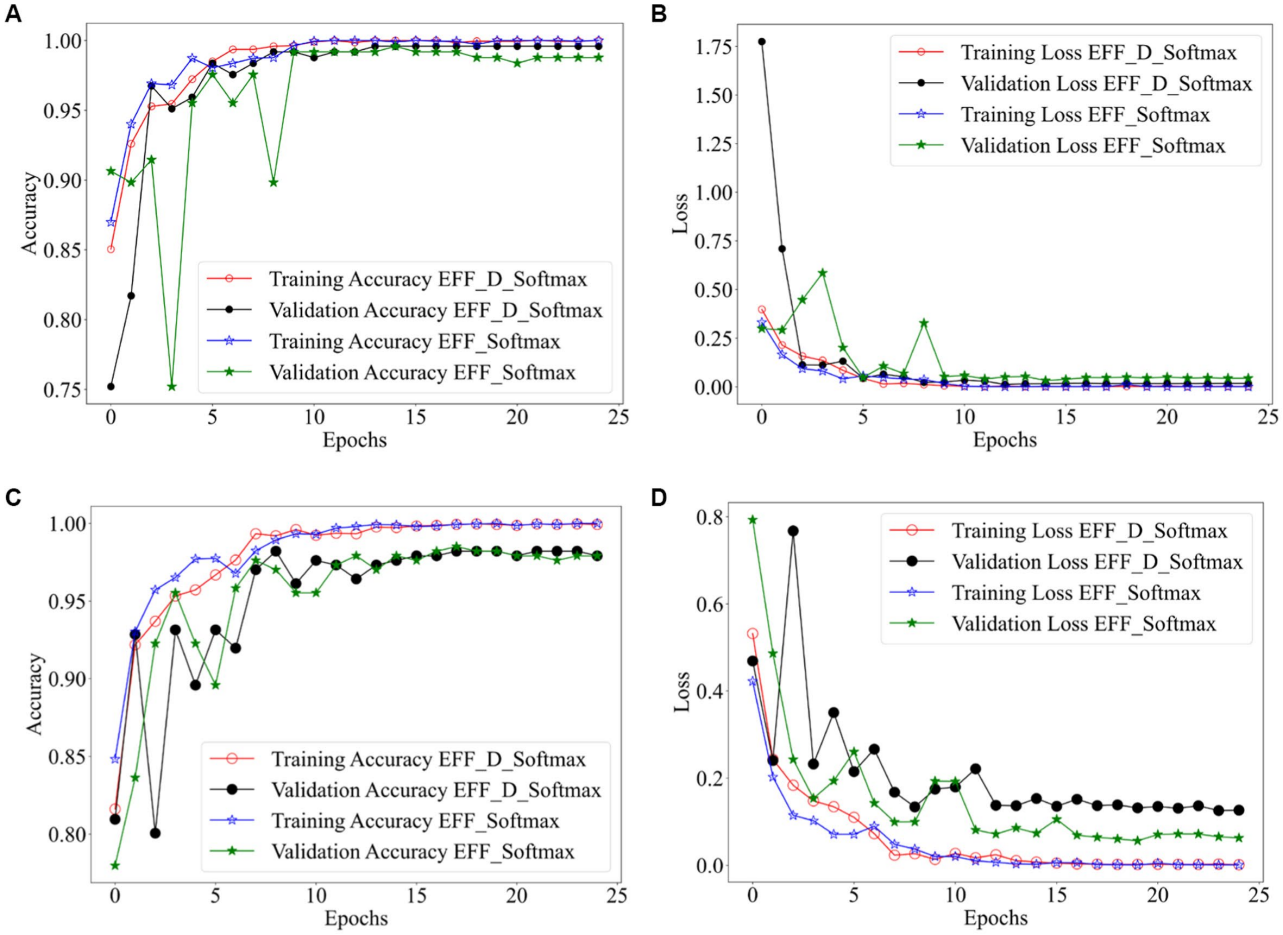
Training results for EFF_D_Softmax and EFF_SoftMax, **(A)** accuracy curve (Chen dataset) **(B)** loss curve (Chen dataset) **(C)** accuracy curve (Kaggle dataset) **(D)** loss curve (Kaggle dataset).

Figure 2 shows the training results of the EFF_D_Softmax model and the EFF_Softmax model on the training sets of both datasets. Images in Figures 2A,B depict the training results obtained from the Chen dataset, while images in Figures 2C,D represent the training outcomes achieved using the Kaggle dataset. In relation to the Chen dataset, the EFF_D_Softmax model demonstrates an accuracy of 100 and 99.59% on the training and validation sets, respectively. Similarly, the EFF_Softmax model achieves accuracies of 100 and 99.18% on the training and validation sets, respectively. For the Kaggle dataset, the EFF_D_Softmax model achieves 99.93 and 98.21% accuracy on the training and validation sets, respectively. Similarly, the EFF_Softmax model achieves 100 and 98.51% accuracy on the training and validation sets, respectively.

**Figure 3:**
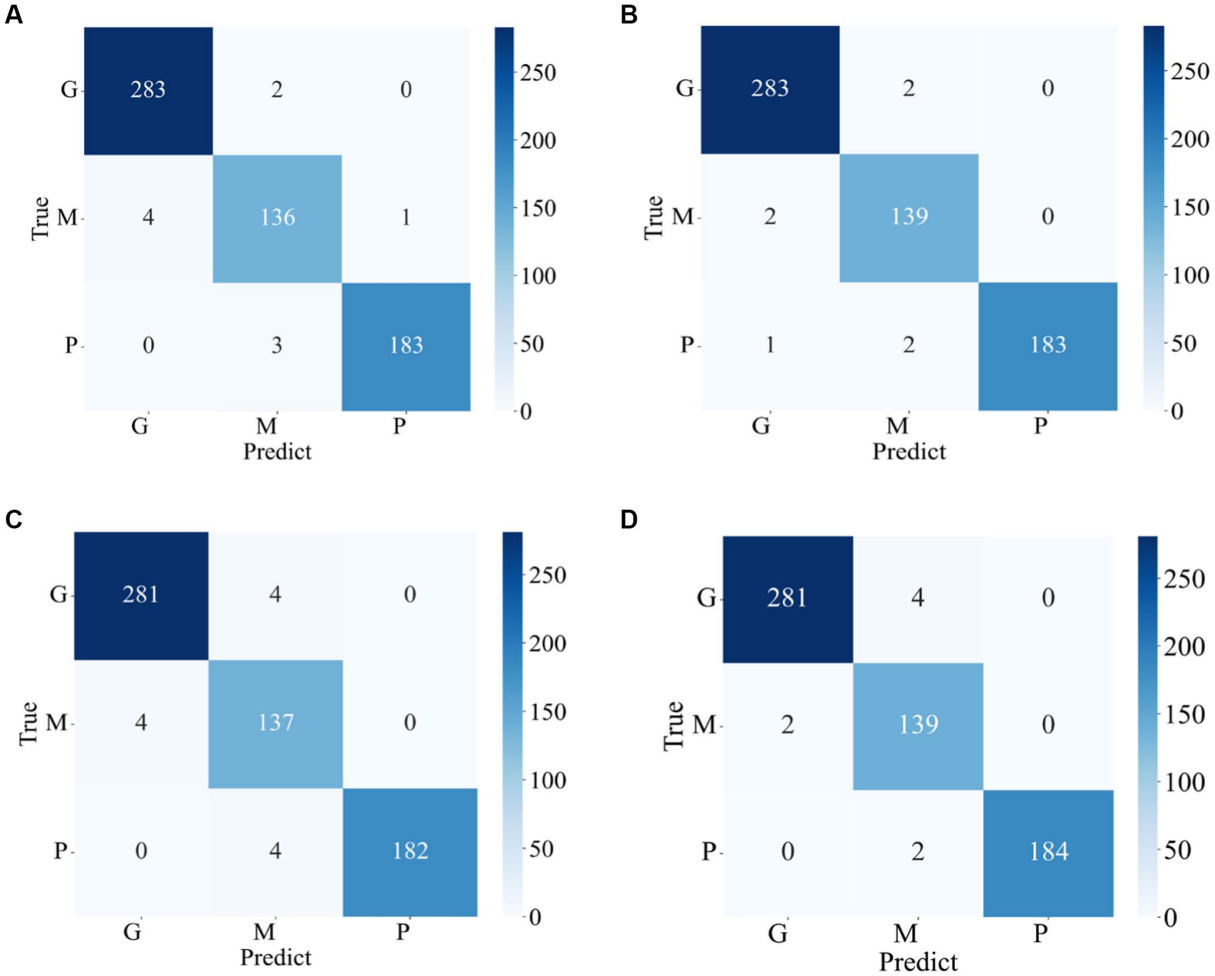
Confusion matrix of the proposed model in the Chen **(A)** EFF_D_Softmax **(B)** EFF_D_SVM **(C)** EFF_Softmax **(D)** EFF_SVM.

**Figure 4:**
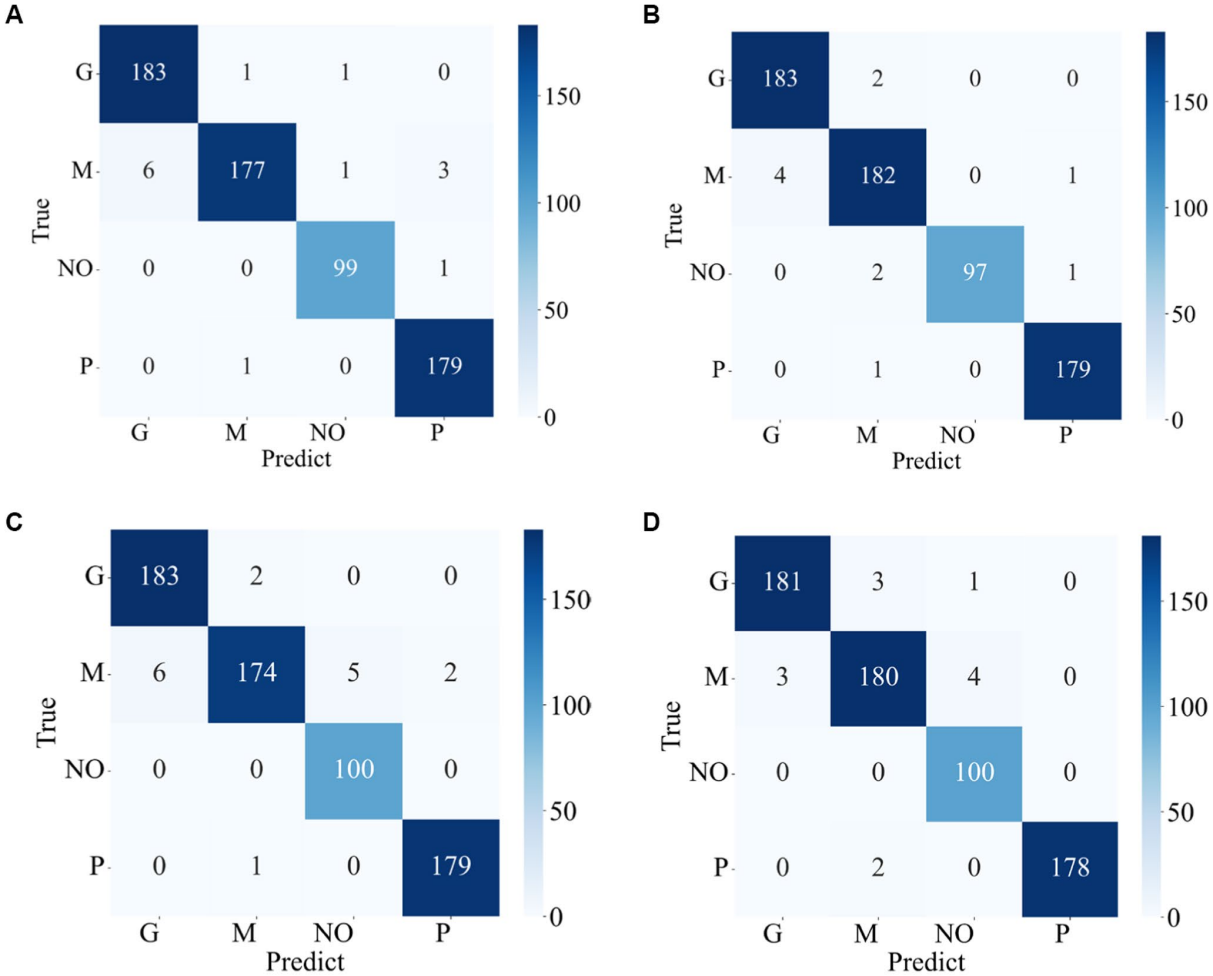
Confusion matrix of the proposed model in the Kaggle **(A)** EFF_D_Softmax **(B)** EFF_D_SVM **(C)** EFF_Softmax **(D)** EFF_SVM.

**Figure 5:**
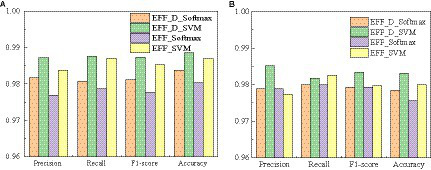
Average metrics for classification results **(A)** Chen **(B)** Kaggle.

The confusion matrixes for the classification results of the proposed method are shown in Figures 3, 4. Eqs 6–9 are utilized to calculate the detailed values of the model classification results from the confusion matrixes. The labels G, M, P, and NO represent different types of brain tumors: G for glioma, M for meningioma, P for pituitary tumor, and NO for the absence of a tumor. The obtained model metrics on the Chen and Kaggle are listed in Tables 3, 4, respectively. Moreover, to visually show the superiority of the adopted EFF_D_SVM model, the average metrics for classification results on the Chen dataset and Kaggle dataset are shown in Figures 5A,B, respectively. On the Chen, EFF_D_SVM showed the best classification results. On the Kaggle, as can be seen from Figure 5B, EFF_D_SVM outperformed the other models in terms of accuracy, f1-score and precision, but its recall rate was lower than that of EFF_SVM. Through the comparison in Table 4, we can see that the recall rate of EFF_D_SVM was higher than that of EFF_SVM for glioma, meningioma, and pituitary, and slightly lower than that of the EFF_SVM for no tumor. In a comprehensive analysis, the classification ability of EFF_D_SVM is still better than that of EFF_SVM. The Softmax classifier constantly strives for higher probabilities for correct classifications and lower probabilities for incorrect classifications, aiming to minimize the loss value. In contrast, the SVM classifier only needs to satisfy the boundary value and does not need to perform subtle manipulations on the concrete scores. Consequently, the Softmax classifier exhibits overfitting in brain tumor classification. Typically, Softmax is employed for large datasets, while SVM is suited for smaller datasets. In this paper, a small dataset is used, which could also contribute to the favorable performance of SVM classification.

**Table 3:** Detailed metrics values of the proposed model on the Chen dataset.

Proposed model	Tumor type	Precision (%)	Recall (%)	F1-score (%)	Accuracy (%)
EFF_D_Softmax	Glioma	98.61	99.30	98.95	98.37
	Meningioma	96.45	96.45	96.45	
	Pituitary	99.46	98.39	98.92	
	**Average**	**98.17**	**98.07**	**98.11**	
EFF_D_SVM	Glioma	98.95	99.30	99.12	98.86
	Meningioma	97.20	98.58	97.89	
	Pituitary	1.00	98.39	99.19	
	**Average**	**98.72**	**98.76**	**98.73**	
EFF_Softmax	Glioma	98.60	98.60	98.60	98.04
	Meningioma	94.48	97.16	95.80	
	Pituitary	1.00	97.85	98.91	
	**Average**	**97.69**	**97.87**	**97.77**	
EFF_SVM	Glioma	99.29	98.60	98.94	98.69
	Meningioma	95.86	98.58	97.20	
	Pituitary	1.00	98.92	99.46	
	**Average**	**98.38**	**98.70**	**98.53**	

**Table 4:** Detailed metrics values of the proposed model on the Kaggle dataset.

Proposed model	Tumor type	Precision (%)	Recall (%)	F1-score (%)	Accuracy (%)
EFF_D_Softmax	Glioma	96.83	98.92	97.86	97.85
	Meningioma	98.88	94.65	96.72	
	No Tumor	98.02	99	98.51	
	Pituitary	97.81	99.44	98.62	
	Average	97.89	98	97.93	
	Meningioma	98.88	94.65	96.72	
	No Tumor	98.02	99	98.51	
	Pituitary	97.81	99.44	98.62	
	**Average**	**97.89**	**98**	**97.93**	
EFF_D_SVM	Glioma	97.86	98.92	98.39	98.31
	Meningioma	97.33	97.33	97.33	
	No Tumor	1	97	98.48	
	Pituitary	98.9	99.44	99.17	
	Meningioma	97.33	97.33	97.33	
	No Tumor	1	97	98.48	
	Pituitary	98.9	99.44	99.17	
	**Average**	**98.52**	**98.17**	**98.34**	
EFF_Softmax	Glioma	95.31	98.92	97.08	97.55
	Meningioma	98.31	93.05	95.6	
	No Tumor	96.15	1	98.04	
	Pituitary	1	99.44	99.72	
	**Average**	**97.88**	**98**	**97.93**	
EFF_SVM	Glioma	98.37	97.84	98.1	98
	Meningioma	97.3	96.26	96.77	
	No Tumor	95.24	1	97.56	
	Pituitary	1	98.89	99.44	
	**Average**	**97.73**	**98.25**	**97.97**	

**Figure 6:**
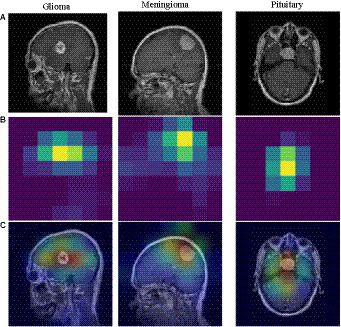
Grad-CAM visualization of different tumors. **(A)** brain tumor **(B)** heatmap **(C)** superimposed image.

On one hand, the model’s fitting ability pertains to its capacity to accurately capture patterns and relationships within the training data. On the other hand, generalizability encompasses the model’s capability to perform with data which has not encountered previously. When too much emphasis is placed on the model’s ability to fit, the model may overfit on the training data set and underperform on new data not seen before. Moreover, as can be observed from Figures 2, the EFF_D_Softmax and EFF_Softmax fit well on the training sets of both datasets. However, model validation on test sets for both datasets found that the EFF_D_Softmax outperformed the EFF_Softmax. Therefore, EFF_D_Softmax has better anti-fitting and generalization ability.

**Figure 7:**
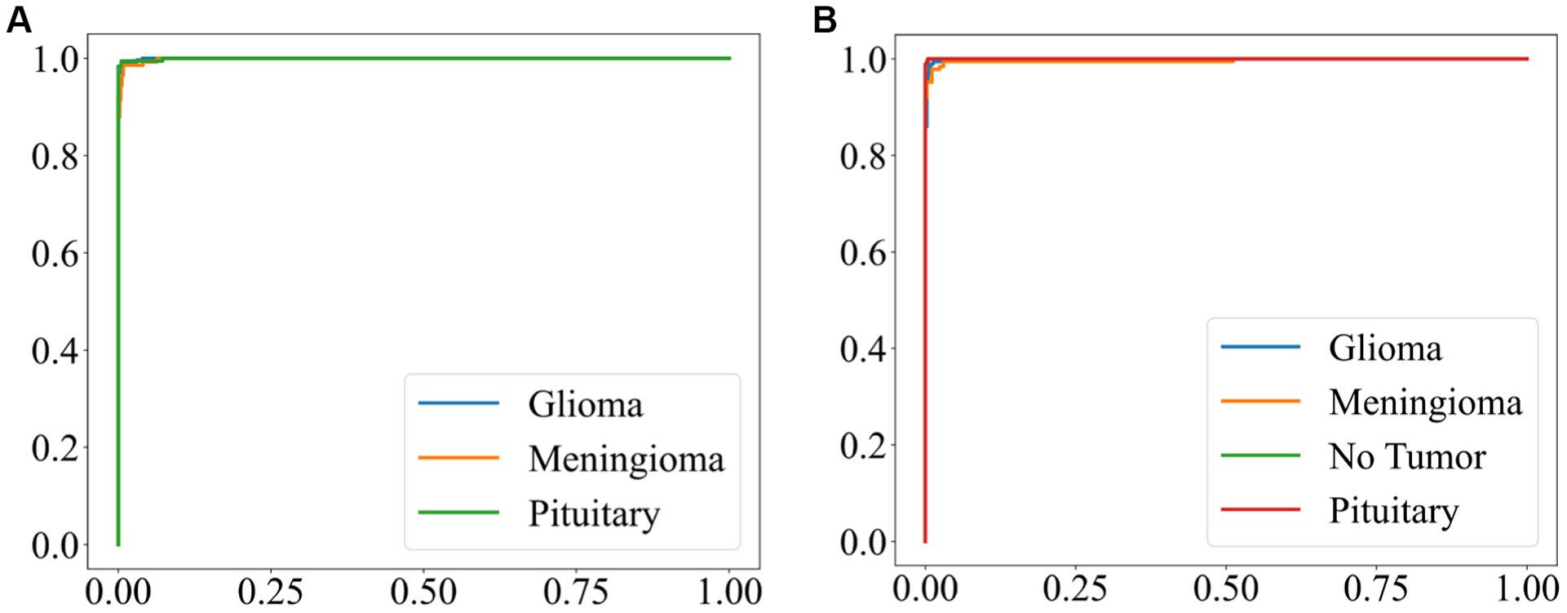
ROC curve for EFF_D_SVM **(A)** Chen **(B)** Kaggle.

The Receiver Operating Characteristic (ROC) curve offers an effective tool to assess the model classification ability by the relationship curve between the false positive rate and the true positive rate. The Area Under the Curve (AUC) provides essential information about the ability of the proposed model to differentiate between tumor types. The classifier performance is better if the AUC value is higher. The ROC curves of EFF_D_SVM for Chen and Kaggle are depicted in Figures 7A,B, respectively. These curves, which are very close to the upper-left corner, indicate that the EFF_D_SVM model has excellent classification ability. In the Chen dataset, the AUC values of EFF_D_SVM for glioma, meningioma, and pituitary are 0.9994, 0.9998, and 0.9996, respectively. And in the Kaggle dataset, the AUC values of EFF_D_SVM for glioma, meningioma, pituitary adenoma and tumor-free are 0.9937, 0.9964, 0.9999 and 0.9999, respectively.

The classification results obtained by our proposed model are compared with those obtained by previous state-of-the-art models that used the same dataset, as shown in Table 5. It can be observed that the proposed model outperforms the available state-of-the-art methods, both on Chen and Kaggle datasets. In particular, the accuracy of our proposed EFF_D_SVM model achieve 98.86 and 98.31% on Chen and Kaggle, respectively.

**Table 5:** Comparison of our proposed model with previous models.

Reference	Dataset	Method	Accuracy(%)	F1-score(%)	Precision(%)	Recall (%)
Swati et al. (2019)	Chen	Fine-tuned VGG19	94.82	91.73	89.52	94.25
Sekhar et al. (2022)	Chen	GoogleNet+KNN	98.3	97.24	97.24	97.23
Öksüz et al. (2022)	Chen	ResNet18 + ShallowNet+SVM	97.25	95.26	95.25	95.27
Satyanarayana (2023)	Chen	DCNN-MCN	94	–	–	–
Deepak and Ameer (2023)	Chen	Majority voting	95.6	–	–	–
Jaspin and Selvan (2023)	Chen	MCCNN	95.17	95	96	95
Mehnatkesh et al. (2023)	Chen	Optimizing ResNet	98.694	98.458	98.53	98.40
Kang et al. (2021)	Kaggle	MobileNetV2 + SVM	98.16	–	–	–
Muezzinoglu et al. (2023)	Kaggle	PatchResNet	98.1	98.1	97.91	98.15
Alanazi et al. (2022)	Chen	22-layer-CNN	96.89	–	–	–
	Kaggle		95.75	–	–	–
Kibriya et al. (2022)	Chen	13-layer CNN	97.2	-	97	96
	Kaggle		96.9	–	–	–
Proposed model	Chen	EFF_D_SVM	98.86	98.73	98.76	98.72
	Kaggle		98.31	98.34	98.52	98.17

To understand the model’s area of interest for a category, we visualized it using the Grad-CAM (Selvaraju et al., 2020) technique. This technique can help us to understand how the model distinguishes different types of brain tumors. In this paper, Grad-CAM is used to create a class activation heat map. The contribution of a specific part in differentiating between different brain tumors is directly proportional to the darkness of its corresponding color. Figure 6 shows a visual depiction of EFF_D_SVM for brain tumor image categorization using Grad-CAM. The heat map produced by Grad-CAM is displayed in Figure 6B, while Figure 6C exhibits the outcome of superimposing the heat map onto the original image. Figure 6C visually demonstrates the application of the grad-cam technique, where the area of the brain tumor is highlighted in red. This indicates that the tumor region serves as a prominent feature in differentiating brain tumors, although the surrounding area is also included.

### 3.4. Cross-dataset validation and robustness validation

To further demonstrate the robustness of our proposed model, cross-validating experiment on multiple datasets was also carried out. Considering that the Chen dataset is three-class dataset while the Kaggle dataset comprises four classes, EFF_D_SVM and EFF_SVM will be evaluated on Kaggle while excluding the normal category classes. This decision was made to ensure the model reliability and validity while avoiding any potential confounding factors. Table 6 es the results of cross-dataset validation. EFF_D_SVM achieves an F1-score of 97.61% and accuracy of 97.62%, which performs better than other models. These results suggest that the proposed EFF_D_SVM model has strong robustness.

**Table 6:** Results of cross-data validation.

Model	Precision (%)	Recall (%)	F1-score (%)	Accuracy (%)
EFF_D_Softmax	97.20	97.08	97.08	97.09
EFF_D_SVM	97.67	97.61	97.61	97.62
EFF_Softmax	92.67	94.37	93.42	94.04
EFF_SVM	97.44	97.37	97.36	97.37

**Figure 8:**
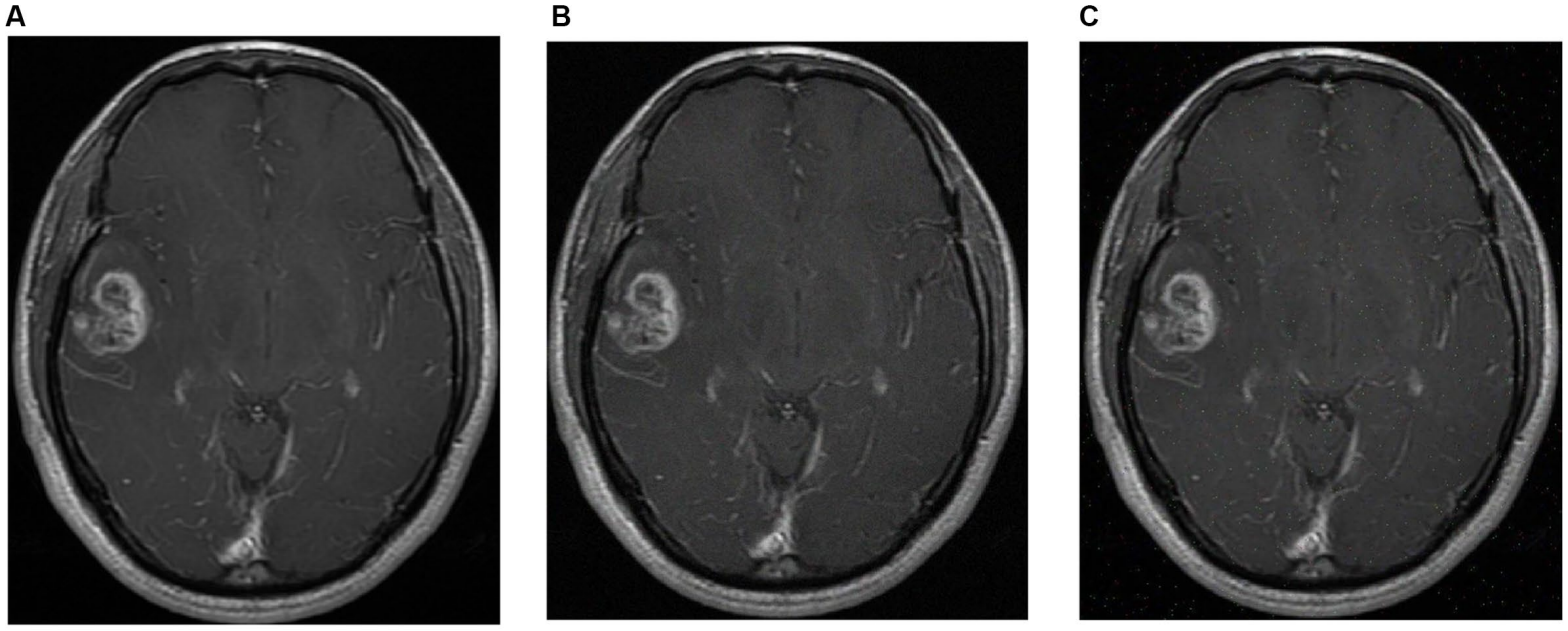
Images after adding noise **(A)** original image **(B)** Gaussian noise **(C)** S&P.

To further evaluate the robustness of the model, gaussian noise and S&P noise were added to the test sets of brain tumor images, respectively. Gaussian noise constitutes a form of noise characterized by a probability density function that adheres to a Gaussian distribution. This type of noise frequently manifests in digital images. The emergence of Gaussian noise stems from intricate interplays among circuit components, prolonged functioning of the image sensor, and various other contributing factors. S & P is often referred to as impulse noise, which randomly modifies certain pixel values to appear as sporadic black-and-white dots in the image. This form of noise arises from the image sensor, transmission channel, decoding, and processing stages, resulting in both bright and dark dots scattered throughout the image. The robustness of models was verified by adding noise to datasets. Here, the variance of the Gaussian noise has been configured at 0.001, while the S&P noise affects 0.005 of the total pixels. Subsequently, the resulting image, which encompasses both Gaussian and S&P noise, is visually depicted in Figure 8. The Table 7 reveals that EFF_D_SVM demonstrates superior robustness compared to the other three models. Following the introduction of Gaussian and S&P noise to the images, EFF_D_SVM achieves classification accuracies of 95.42 and 96.24% for the Chen dataset, and 93.40 and 95.71% for the Kaggle dataset. Notably, for the test set of the Kaggle dataset, both EFF_Softmax and EFF_SVM exhibit classification accuracies below 90% upon the introduction of Gaussian noise, which shows that they have weak robustness.

**Table 7:** Classification results of models after adding noise.

Dataset	Type of noise	Model	Precision (%)	Recall (%)	F1-score (%)	Accuracy (%)
Chen	Gaussian noise	EFF_D_Softmax	94.96	94.23	94.36	94.44
		EFF_D_SVM	95.76	95.17	95.34	95.42
		EFF_Softmax	93.46	89.49	90.42	92.32
		EFF_SVM	93.69	89.31	90.57	92.16
	Salt and pepper noise	EFF_D_Softmax	95.73	94.81	95.10	95.42
		EFF_D_SVM	96.67	95.22	95.83	96.24
		EFF_Softmax	95.73	94.81	95.10	95.42
		EFF_SVM	94.58	93.16	93.45	94.28
Kaggle	Gaussian noise	EFF_D_Softmax	94.14	92.89	93.25	93.10
		EFF_D_SVM	93.76	93.25	93.54	93.40
		EFF_Softmax	84.62	85.47	83.40	83.44
		EFF_SVM	86.86	88.41	86.12	86.81
	Salt and pepper noise	EFF_D_Softmax	94.38	95.04	94.45	94.33
		EFF_D_SVM	95.99	96.25	96.06	95.71
		EFF_Softmax	92.89	93.05	92.31	92.02
		EFF_SVM	91.09	91.93	91.14	90.95

## 4. Conclusion

Early diagnosis of brain tumors is critical for selecting appropriate treatment options and saving the lives of patients. The manual examination of brain tumors is a laborious and time-consuming process, therefore, it is necessary to develop an automated detection method to aid physicians. This paper proposes a novel approach to detect multiple types of brain tumors. In this paper, a new feature extraction module EEF_D is proposed. Features are extracted from brain tumor images using EFF_D and the features are classified using SVM. To verify the effectiveness of our approach, a series of comparative experiments were also performed. The EFF_D_SVM model exhibits excellent classification ability for brain tumors with minimal Data pre-processing, as validated on both the Chen and Kaggle datasets. On the Chen dataset, EFF_D_SVM achieves a classification accuracy of 98.86% and an F1-score of 98.73%, and on the Kaggle dataset, it yields the corresponding values of 98.31 and 98.34%, respectively. Through comparison with other state-of-the-art models, the proposed model outperforms the available state-of-the-art methods. Moreover, by means of cross-validation experiments, the proposed model is proved to be very robust. In future work, samples from other types of brain disorders could be added to expand the dataset to improve the performance of the model, in turn to enhance the ability to identify other disorders.

## Data availability statement

The datasets presented in this study can be found in online repositories. The names of the repository/repositories and accession number(s) can be found below: https://figshare.com/articles/dataset/brain_tumor_dataset/1512427 https://www.kaggle.com/datasets/sartajbhuvaji/brain-tumor-classification-mri.

## Author contributions

JZ: Investigation, Methodology, Project administration, Writing – original draft. XT: Software, Writing – original draft. WC: Writing – review & editing, Formal analysis, Software, Validation, Visualization. GD: Writing – review & editing, Project administration, Supervision. QF: Writing – review & editing, Validation. HZ: Investigation, Writing – review & editing. HJ: Investigation, Validation, Writing – review & editing.

## Footnotes

^1^ tf.keras.applications.efficientnet.EfficientNetB0 | TensorFlow v2.12.0 (google.cn)
